# The overlooked link between reproductive system disorders and depression: a cohort study in 2 million women

**DOI:** 10.1017/S0033291725102602

**Published:** 2025-11-21

**Authors:** Mette Bliddal, Rikke Wesselhoeft, Lotte Rasmussen, Magdalena Janecka, Nina Zaks, Lone Kjeld Petersen, Sofie Egsgaard, Peter Bjødstrup Jensen, Trine Munk-Olsen

**Affiliations:** 1 Department of Clinical Research, University of Southern Denmark: Syddansk Universitet, Odense, Denmark; 2 Child and Adolescent Psychiatry Southern Denmark, Mental Health Services in the Region of Southern Denmark, Odense, Denmark; 3 Clinical Pharmacology, Pharmacy and Environmental Medicine, Department of Public Health, University of Southern Denmark, Odense, Denmark; 4 Department of Child and Adolescent Psychiatry, New York University Grossman School of Medicine, New York, NY, USA; 5 Department of Population Health, New York University Grossman School of Medicine, New York, NY, USA; 6 Department of Obstetrics and Gynecology, Odense University Hospital, Odense, Denmark

**Keywords:** depression, endometriosis, female, irregular menstruation, menstrual pain, mental health, pain, polycystic ovary syndrome, reproductive disorders, reproductive system disorders, women

## Abstract

**Background:**

Depression rates are higher in women, especially during periods of hormonal fluctuation. Reproductive system disorders (RSDs), which often disrupt hormonal balance, may contribute to this mental health burden. Despite their prevalence and significant health implications, the link between RSDs and depression remains underexplored, leaving a gap in understanding these women’s mental health risks.

**Methods:**

Using Danish nationwide health registers (2005–2018), we conducted a cohort study of 2,295,824 women aged 15–49, examining depression outcomes in 265,891 women diagnosed with 24 RSDs, including endometriosis, polycystic ovary syndrome, and pain-related diagnoses. For each RSD, age-matched controls were selected. We calculated incidence rates, incidence rate ratios, and prevalence proportions of depression diagnoses or antidepressant use around RSD diagnosis.

**Results:**

Across all RSD subtypes, women demonstrated higher rates of depression both before and after diagnosis, with a peak within the year following diagnosis. Incidence rate ratios within 1 year of RSD diagnosis ranged from 1.15 (95% confidence interval [CI] 1.06–1.25) to 2.09 (95% CI 1.98–2.21), depending on RSD subtype. Elevated depression prevalence was observed 3 years before diagnosis, suggesting mental health impacts may have preceded clinical RSD identification.

**Conclusions:**

This study reveals a striking association between RSDs and depression. Women with RSDs are more likely to suffer from depression, before and after RSD diagnosis, highlighting the need for integrated mental health screening and intervention. With over 10% of women affected by RSDs, addressing this overlooked mental health burden is imperative for improving well-being in a significant portion of the population.

## Introduction

Women have consistently been shown to have a twofold higher risk of depression compared to men (Kuehner, 2017; Pedersen et al., 2014), with this risk particularly elevated during periods of hormonal fluctuation, such as pregnancy, the postpartum period, and menopause (Lokuge et al., 2011; Palomba et al., 2018). Reproductive system disorders (RSDs), including conditions such as endometriosis, polycystic ovary syndrome (PCOS), and disorders involving abnormal bleeding and pain, affect a significant portion of women (Cooney, Lee, Sammel, & Dokras, 2017; Estes et al., 2021; Fortin, Flyckt, & Falcone, 2018). While the etiology of these disorders varies, they are uniformly associated with poor mental health and decreased quality of life (Cooney et al., 2017; Estes et al., 2021; Fortin et al., 2018). Many RSDs are also associated with reduced fertility, further exacerbating the risk of depression (‘Infertility’, n.d.). In addition, imbalances in sex hormones, a key feature of many RSDs, can directly influence mental health, contributing to higher rates of depression among affected women (Kundakovic & Rocks, 2022). These intertwined relationships between reproductive disorders and mental well-being underscore the importance of a comprehensive understanding of these conditions.

Despite the clear association between RSDs and mental health challenges, the relationship between RSDs and depression in women of reproductive age remains poorly understood. Both RSDs and depressive disorders are frequently characterized by considerable diagnostic delays, which complicated the establishment of temporal patterns or causality. While much of the existing research has focused on specific conditions, particularly PCOS, there is a need for broader investigations into the mental health impact of a wider range of RSDs (Zaks et al., 2023). Moreover, previous studies have not fully explored the potential bidirectionality of the relationship between RSDs and depression, leaving gaps in our understanding of these correlations.

To address these gaps, we aimed to investigate the incidence and prevalence of depression in women of reproductive age before and after their incident diagnosis of RSDs. Our study provides a comprehensive examination of depression risk surrounding the diagnosis of 24 different RSDs, utilizing a nationwide cohort to assess the mental health impact across a large population.

## Methods

We conducted a descriptive cohort study using nationwide health registers in Denmark. The primary aim was to characterize the temporal cooccurrence between RSDs and depression. The study included all women with any of the 24 main RSDs, classified by third level of the International Classification of Diseases, version 10 (ICD-10), between 2005 and 2018. We examined the incidence rates and prevalence proportions of depression, defined either by a registered hospital diagnosis or the filling of any antidepressant prescription within a 3-year period before and after the first diagnosis of any RSD. Age-matched women without an RSD diagnosis served as comparators.

### Data sources

Denmark’s tax-funded healthcare system provides universal coverage for all residents, and each individual is assigned a unique personal identification number. This number enables linkage across all healthcare and administrative registers (Morten Schmidt et al., 2019). Since 1995, the Danish National Patient Register (Patient Register) has recorded all in- and outpatient hospital contacts, including psychiatric services and emergency room visits (Morten Schmidt et al., 2015). The Patient Register contains information on the date of contact and assigned diagnoses according to ICD-10 (‘WHO | ICD-10 Online Versions’, n.d.). The Danish National Prescription Registry (Prescription Registry) contains information on all prescriptions filled at outpatient pharmacies since 1995(Pottegård et al., 2017), using the WHO Anatomical Therapeutic Chemical (ATC) Classification system (Norwegian Institute of Public Health, n.d.). This registry includes data on dispensing dates, ATC codes, and treatment indications with high validity (Harbi & Pottegård, 2024; Pottegård et al., 2017). The Danish Civil Registration System provides data on date of death and migration (Schmidt, Pedersen, & Sorensen, 2014).

### Population

We identified all women aged 15–49 years in Denmark with an incident diagnosis of any RSD from January 1, 2005 to December 31, 2018, using the Patient Register (Morten Schmidt et al., 2015). RSDs included inflammatory diseases of the female pelvic organs (ICD-10 N70–77), noninflammatory disorders of the female genital tract (N80–94), and ovarian dysfunction (E28) (Zaks et al., 2023). The disorders were categorized at the third level of the ICD-10 classification, resulting in 24 distinct categories, each with its own subcategories (see ST 1 for the full list of categories and subcategories). We excluded conditions related to pregnancy and childbirth (e.g. abortion, infertility), as we hypothesized these events may have distinct etiological relationships with mental health.

We constructed an overall RSD cohort and 24 subcohorts for each third-level ICD-10 RSD diagnosis category. Both primary and secondary diagnosis codes were used to define case status. Women were considered incident cases in the RSD overall cohort upon their first-ever diagnosis of any RSD, and incident cases in the specific RSD subcohorts were based on the *first* diagnosis of that RSD. An 8-year run-in period was applied to exclude women who immigrated less than 8 years prior to their first RSD diagnosis. In the overall RSD analyses, women could have incident diagnoses in multiple disorder categories but were counted only once. The date of the first diagnosis was defined as the index date. For each RSD case within each disorder category, five women without the index diagnosis were randomly selected from the general female population (matched on birth month and year) using risk set sampling (SF 1). These women formed the comparison group, with the same 8-year run-in period applied.

### Definition of depression and period of interest

For the main analyses, we defined depression as either (1) a primary or secondary diagnosis of depressive disorder (ICD-10 code F32–39) during an inpatient or outpatient hospital contact or (2) the filling of a prescription of antidepressants (ATC code N06A). Since most women with depressive disorders are diagnosed and treated in general practice or by privately practicing psychiatrists (Musliner et al., 2019) and may not be captured in the Patient Register, antidepressant prescriptions serve as a proxy measure for a depressive disorder. Depression was tracked from 3 years before to 3 years after the index date (day of RSD diagnosis) until the date of death, migration, or 3 years after the index date, whichever occurred first. Women in the comparator group were also censored upon an RSD diagnosis.

Incident depression was defined as the first depression diagnosis or antidepressant prescription in women with no such history in the preceding 5 years. Once a woman was identified as having incident depression, she was considered a prevalent case for the remainder of the study period.

### Statistical analyses

We conducted the following analyses for each of the 24 level 3 ICD-10 RSD categories, as specified in Table 1, and for the age-matched comparison groups.

**Table 1.** Number of incident cases of women with a reproductive system disorder overall and by diagnosis group in Denmark (2000–2018)

Disorders	ICD–10 codes^a^	N	%^b^	Median age at onset (IQR^c^)
Excessive, frequent, and irregular menstruation	N92	98,607	37	42 (36–46)
Noninflammatory disorders of ovary, fallopian tube, and broad ligament	N83	37,741	14	36 (27–43)
Dysplasia of cervix uteri	N87	34,992	13	31 (26–39)
Pain and other conditions associated with female genital organs/menstrual cycle	N94	33,312	13	33 (24–41)
Polyp of female genital tract	N84	22,476	8.5	42 (37–46)
Other abnormal uterine and vaginal bleeding	N93	18,777	7.1	33 (26–41)
Endometriosis	N80	18,525	7.0	35 (28–42)
Female genital prolapse	N81	16,337	6.1	40 (33–46)
Ovarian dysfunction, including polycystic ovary syndrome	E28	15,302	5.8	27 (23–32)
Other inflammation of vagina and vulva	N76	12,926	4.9	32 (24–41)
Inflammatory disease of uterus, except cervix	N71	8965	3.5	31 (25–38)
Salpingitis and oophoritis	N70	8863	3.4	33 (24–41)
Disease of Bartholin’s gland	N75	7311	2.9	34 (27–41)
Other noninflammatory disorders of vulva and perineum	N90	7192	2.7	32 (24–40)
Ascent, scanty, and rare menstruation	N91	6133	2.3	26 (19–34)
Other noninflammatory disorders of vagina	N89	3878	1.5	33 (25–41)
Other female pelvic inflammatory diseases	N73	3696	1.4	35 (28–42)
Other noninflammatory disorders of uterus, except cervix	N85	3290	1.2	42 (36–46)
Inflammatory disease of cervix uteri	N72	1833	0.69	33 (25–40)
Other noninflammatory disorders of cervix uteri	N88	1729	0.65	37 (31–43)
Erosion and ectopia of cervix uteri	N86	1636	0.62	33 (26–40)
Fistulae involving female genital tract	N82	902	0.33	39 (30–45)
Vulvovaginal ulceration and inflammation in diseases classified elsewhere	N77	640	0.24	28 (22–37)
Female pelvic inflammatory disorders in diseases classified elsewhere	N74	202	0.08	22 (18–28)
Any reproductive system disorders	E28, E70-E77, N80-N94	265,891	100	36 (27–43)

*Note:* Numbers do not add up as one individual could be diagnosed with more than one disorder.

a Including subcategories.

b Percentage of all reproductive system disorders.

c Interquartile range.

First, we calculated the quarterly incidence rates of depression from 3 years before to 3 years after the index date (day of incident RSD diagnosis). Time was split into yearly quartiles to describe detailed trajectories. The numerator was the number of incident depression cases per quarter, and the denominator was the total risk time contributed by the population in that quarter, measured in ‘person-quartiles’ (91 days).

We applied locally estimated scatterplot smoothing, a nonparametric method that uses local regression to fit a smooth curve through the data points (Cleveland & Devlin, 1988). Notably, there was a general trend of decreasing depression incidence in the total population during the study period.

Second, we calculated 2-year incidence rates of depression from 1 year before to 1 year after the index date, keeping person-quartiles as a time measure for comparison. We then calculated the depression incidence rate ratio for each RSD category by comparing the rates in RSD patients with those in the age-matched comparison group.

Third, we described the quarterly prevalence of depression from 3 years before to 3 years after the index date by dividing the number of women with prevalent depression in each quarter by the total number of women contributing time in that quarter. Women diagnosed with incident depression prior to the study period were considered prevalent cases throughout the study period.

### Supplementary analyses

We performed additional analyses to refine our findings. First, we repeated the incidence and prevalence analyses, restricting antidepressant prescriptions to those specifically indicated for depression, to exclude prescriptions for other indications. Second, we restricted the analyses to hospital-diagnosed depression cases to identify women with more severe depression.

All analyses were conducted using R, version 4.3.2.

## Results

Among the 2,295,824 women aged 15–49 years in Denmark from 2005 to 2018, we identified 265,891 women (12%) with at least one registered RSD (Table 1). Below, we present results for the most prevalent RSDs with sample sizes greater than 15,000 (a total of nine subcohorts). Results for the overall RSD cohort and the remaining 15 specific RSDs are available in the Supplementary Material.

The most frequently diagnosed RSD category was *excessive, frequent, and irregular menstruation* (n = 98,607, 37% of all RSDs), followed by *noninflammatory disorders of the ovary, fallopian tube, and broad ligament* (n = 37,741, 14%) and *dysplasia of the cervix uteri* (n = 34,992, 13%). *Endometriosis* and *ovarian dysfunction, including* PCOS, were diagnosed in 18,525 (7.0%) and 15,302 (5.8%) individuals, respectively. The median age at incident RSD diagnosis varied by condition, ranging from 22 years (interquartile range 18–28 years) for women with *female pelvic inflammatory disorders in diseases classified elsewhere* to 42 years for those with *excessive, frequent, and irregular menstruation*, *polyp of the female genital tract*, and women with *other noninflammatory disorders of the uterus, except the cervix.*

**Figure 1.**
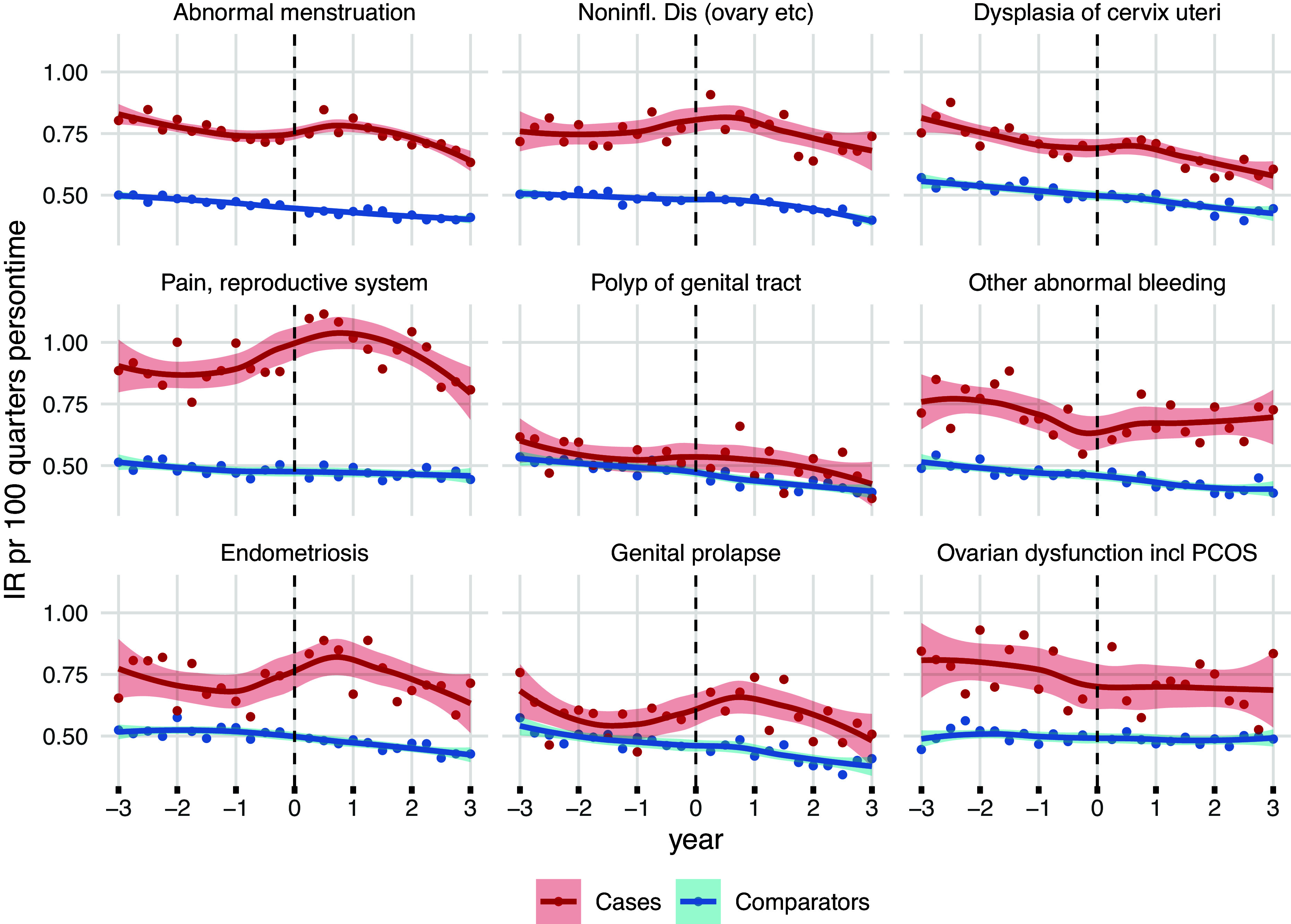
Incidence rate of depression (defined by ICD-10 codes F32–39 or ATC codes N06A) in quarterly intervals, 3 years before and after diagnosis of the nine most common reproductive system disorders (RSDs) (red), compared to age- and calendar-matched women (blue). Panels are presented in order of RSD prevalence (starting upper right): Abnormal menstruation: ICD-10 N92; Noninflammatory disorders: ICD-10 N83; Dysplasia of cervix uteri: ICD-10 N87; Pain, reproductive system: ICD-10 N94; Polyp of genital tract: ICD-10 N84; Other abnormal bleeding: ICD-10 N93; Endometriosis: ICD-10 N80; Genital prolapse: ICD-10 N81; Ovarian dysfunction including polycystic ovary syndrome (PCOS): ICD-10 E28. The dotted lines indicate index date (day of incident RSD diagnosis).

Within the nine most frequent RSD groups, the quarterly incidence rates of depression were higher for women with RSDs compared to the comparator groups during the 3 years before and after the index date (Figure 1). Most RSDs showed elevated depression incidence rates within the first year after the index date, with the highest rates observed in women with *pain and other conditions associated with female genital organs and the menstrual cycle* and in those with *endometriosis.* Similar patterns were observed for the less frequent RSDs (Supplementary Figure 2), although larger variations and wider confidence intervals (CIs) were noted due to fewer observations.

The 2-year depression incidence rates from 1 year before to 1 year after the index date were also increased for the nine most common RSD categories compared to the comparators, with incidence rate ratios ranging between 1.15 (95% CI 1.06–1.25) for *polyp of the female genital tract* to 2.09 (95% CI 1.98–2.21) for *pain and other conditions associated with female genital organs and the menstrual cycle* (Table 2). Similar trends were seen for the less common RSDs, although with wider CIs (Supplementary Table 2).

**Table 2.** Incidence and incidence rate ratio of depression (defined by ICD-10 codes F32–39 or ATC codes N06A) within 12 months before and after the date of incident diagnosis of selected reproductive system disorders (RSDs), among women with the nine most common RSDs and an age-matched comparator group. RSDs are ordered by frequency

Disorders, ICD–10	Depression (N)	Risk time	IR^a^	IRR^b^
Abnormal menstruation, N92				
Comparator	13,388	29,918	0.45	1.00
Case	3943	5209	0.76	1.69 (1.63–1.75)
Noninflammatory disease (ovary, etc.), N83				
Comparator	5645	11,666	0.48	1.00
Case	1673	2104	0.80	1.64 (1.56–1.74)
Dysplasia of cervix uteri, N87				
Comparator	5411	10,856	0.50	1.00
Case	1357	1950	0.70	1.40 (1.32–1.48)
Pain, reproductive system, N94				
Comparator	5029	10,586	0.48	1.00
Case	1781	1792	0.99	2.09 (1.98–2.21)
Polyp of genital tract, N84				
Comparator	3094	6612	0.47	1.00
Case	687	1280	0.54	1.15 (1.06–1.25)
Other abnormal bleeding, N93				
Comparator	2635	5768	0.46	1.00
Case	672	1020	0.66	1.44 (1.32–1.57)
Endometriosis, N80				
Comparator	2852	5732	0.50	1.00
Case	787	1058	0.74	1.50 (1.38–1. .62)
Genital prolapse, N81				
Comparator	2237	4832	0.46	1.00
Case	549	899	0.61	1.32 (1.20–1.45)
Ovarian dysfunction incl. PCOS^c^, N28				
Comparator	2419	4942	0.49	1.00
Case	623	893	0.70	1.42 (1.30–1.56)

a Incidence rate per 100 person quarters person time.

b Incidence rate ratio.

c Polycystic ovary syndrome.

**Figure 2.**
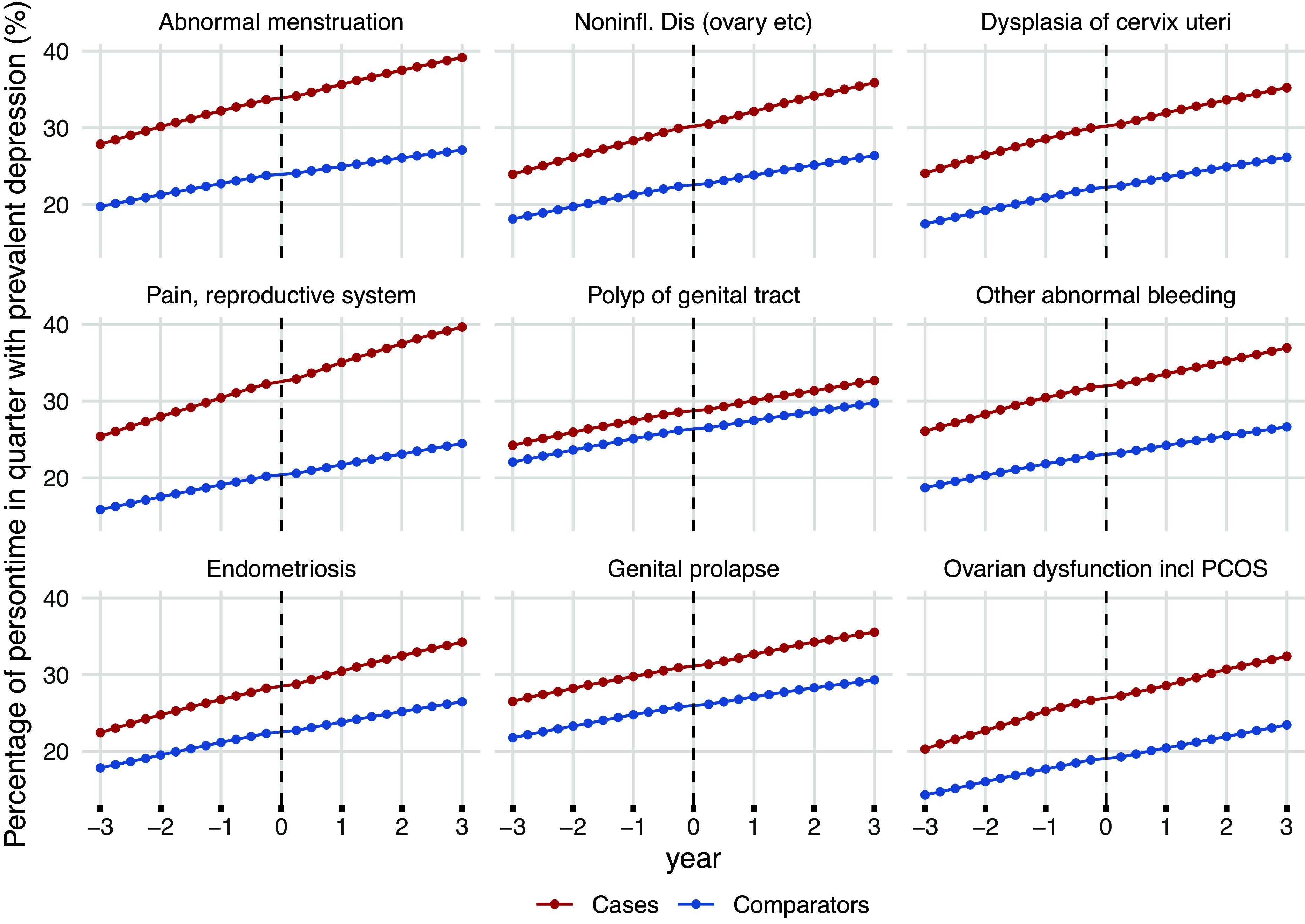
Prevalence proportion of depression (defined by ICD-10 codes F32–39 or ATC codes N06A) in quarterly intervals, 3 years before and after diagnosis of the nine most common reproductive system disorders (RSDs) (red), compared to age- and calendar-matched women (blue). Panels are presented in order of RSD prevalence (starting upper right): Abnormal menstruation: ICD-10 N92; Noninflammatory Disorders: ICD-10 N83; Dysplasia of cervix uteri: ICD-10 N87; Pain, reproductive system: ICD-10 N94; Polyp of genital tract: ICD-10 N84; Other abnormal bleeding: ICD-10 N93; Endometriosis: ICD-10 N80; Genital prolapse: ICD-10 N81; Ovarian dysfunction including polycystic ovary syndrome (PCOS): ICD-10 E28. The dotted lines indicate index date (day of incident RSD diagnosis).

During the entire time window of interest (3 years before and after the index date), the prevalence of depression was higher in women with any of the 24 RSD categories compared to their matched comparators (Figure 2 and Supplementary Figure 3). Notably, the prevalence of depression was already 44% higher in women with any RSD 3 years prior to their incident RSD diagnosis (prevalence ratio in the overall RSD 1.44) compared to the comparators, indicating that the disparity existed well before our study window. Among the nine most common RSDs, the largest initial difference in depression prevalence was seen in women with *pain and other conditions associated with female genital organs and the menstrual cycle, with* a 60% higher prevalence at the index date (prevalence ratio 1.60). The smallest difference was found in women with a *polyp of female genital tract* (prevalence ratio 1.10).

### Supplementary results

When conditioning on antidepressant prescriptions filled solely for depression, the incidence and prevalence trends across the 24 RSD categories were similar to the main results, although the incidence and prevalence estimates of depression were lower (Supplementary Figures 4 and 5).

When restricting the depression definition to hospital-diagnosed depression as a marker of severity, incidence rates decreased as expected, but the differences between women with RSDs and their comparators remained. However, a more pronounced peak in depression incidence rates was observed near the index date for women with RSDs compared to the main analysis, while incidence rates in the comparator group remained stable throughout the time window of interest (Supplementary Figure 6). Similarly, hospital-diagnosed depression prevalence was lower than the broader definition used in the main analysis, but differences in depression prevalence between women with RSDs and their comparators remained evident (Supplementary Figure 7). In cases of rarer RSDs, small sample sizes resulted in wider CIs and imprecise estimates.

## Discussion

In this nationwide study based on Danish register data, we described the incidence and prevalence of depression in women with RSDs compared to age-matched comparators within a 6-year window surrounding their first RSD diagnosis. We hypothesized that RSDs would be associated with depression and, due to the challenges of precisely identifying the onset of both conditions, that associations with depression would be observed both before and after RSD diagnosis.

We found that women with any of the 24 RSD subtypes exhibited a substantially higher risk of depression than the comparators as early as 3 years before their RSD diagnosis. This elevated risk persisted despite suggested differences in both the etiology and biology of these RSDs. The incidence rate of depression remained elevated throughout the 6-year time window observed, indicating that mental well-being is compromised years before their RSD diagnosis and throughout. The difference persisted even when the analyses were restricted to cases where antidepressant prescriptions were specifically for depression, and to severe depression marked by hospital contacts.

Our findings confirm that depression and RSDs frequently co-occur, although determining the exact timing of onset remains difficult. It is well documented that diagnostic delays in chronic RSDs, such as endometriosis, PCOS, and pain-related pelvic disorders, are considerable, with a mean time of symptom onset to diagnosis of 4–11 years (Armour et al., 2020; Pino et al., 2023; Soliman, Fuldeore, & Snabes, 2017). Furthermore, research suggests that women’s self-reported pain related to their reproductive system is often discounted until objective evidence for the cause of pain is identified (Hoffmann & Tarzian, 2001). While we cannot establish causality from our study, it is plausible that delays in RSD diagnosis and treatment gradually impair mental health. Other explanations for the observed cooccurrence may include shared underlying risk factors. For example, previous research has suggested a potential genetic overlap between PCOS and psychiatric disorders, including depression (Cesta et al., 2017; Chen et al., 2024). In addition, common lifestyle-related factors—such as metabolic disturbances and stress —may also contribute to both RSDs and poor mental health. (Gao et al., 2024; Zaks et al., 2023). These findings highlight the importance of accurate and timely diagnosis of RSDs, coupled with an emphasis on mental health care for these women. The observed cooccurrence may also reflect the broader burden of physical comorbidities. Women with chronic or painful RSDs often experience other somatic conditions that can contribute to depressive symptoms through shared biological or psychosocial pathways. These interrelated mechanisms highlight that the association between RSDs and depression likely arises from a complex clustering of physical and mental health factors.

Our results align with previous studies examining specific RSD categories, particularly endometriosis and PCOS, which have consistently demonstrated significant overlap between RSDs and poor mental health (e.g. depression and anxiety) (Muharam et al., 2022; van Barneveld et al., 2022; Zaks et al., 2023). Although the mechanisms linking RSDs and depression are not fully understood, the potential drivers include decreased quality of life as a consequence of RSD symptoms (Muharam et al., 2022; van Barneveld et al., 2022). Additionally, the link between these somatic and psychiatric disorders may involve hormonal changes or dysregulation (Lokuge et al., 2011), and chronic pain, which can impact day-to-day life and exacerbate depressive symptoms (Rayner et al., 2016). For example, alterations in the central nervous system have been observed in women experiencing pain due to endometriosis, possibly contributing to the heightened risk of depression (Bashir et al., 2023; Maulitz et al., 2022). Likewise, prior research has linked premenstrual disorders, included in the RSD category *Pain and other conditions associated with female genital organs and menstrual cycle*, to elevated depression risk,(Liu, Lin, & Zhang, 2024; Wang et al., 2025; Yonkers, O’Brien, & Eriksson, 2008). Consistent with these findings, we found the highest difference in depression risk in women diagnosed with pain-related RSDs, such as *Pain and other conditions associated with female genital organs and menstrual cycle* and *endometriosis.*

The strengths of our study include the use of a nationwide population-based cohort, which provides comprehensive health register data from a tax-funded healthcare system. This ensures full overview of all diagnosed RSDs and complete follow-up and eliminates selection and recall biases (Morten Schmidt et al., 2019). The detailed nature of the register data allowed us to granularly track depression incidence and prevalence in quarterly intervals around the time of RSD diagnosis.

However, our study also has limitations. We used filled prescriptions as a proxy for depression, and while these are generally considered more reliable than medical records or questionnaires (Furu et al., 2010), we cannot confirm that the medications were consumed. Nevertheless, a filled prescription indicates a treatment need assessed by a medical doctor and is indicative of a self-evaluated need. Our supplementary analyses, which excluded antidepressants prescribed for indications other than depression (Harbi & Pottegård, 2024) and focused solely on diagnoses, mirrored the main findings, reinforcing the robustness of our results. Additionally, we cannot rule out referral bias, as women seeking treatment for RSDs may be more likely to have their depression recognized. Due to the nature of the data, we capture RSDs and depression diagnosed in hospital settings and/or depression measured by filled prescriptions, which may limit the generalizability to women with RSDs identified. There is a risk of misclassification of both RSDs and depression due to diagnostic delay and milder cases of either not registered. Finally, we have defined prevalence of depression as any time after an incident depression diagnosis/filled prescription resulting in a heterogeneous group of prevalent depressed. However, having had any a diagnosis of depression is an indicator of vulnerability and risk of recurrence is considerable (Moriarty et al., 2020).

## Conclusion

Depression is more common among women with RSDs compared to women without RSDs, beginning as early as 3 years before the diagnosis and persisting for years afterward. Although the causal relationship between RSDs and depression remains indeterminate, the clear overrepresentation of depression in women with RSDs highlights the need for collaborative approaches between somatic and psychiatric healthcare systems. Integrating mental health screening and care into the treatment of RSDs, and vice versa, should become an essential part of future clinical guidelines.

## Supporting information

Bliddal et al. supplementary material Bliddal et al. supplementary material

## Data Availability

Individual-level data cannot be shared by the authors owing to Danish data protection regulations. Deidentified data can be made available to authorized researchers after application to Forskerservice at the Danish Health Data Authority. Scripts can be acquired upon request. A registered study protocol can be found here: OSF | Depression among women with disorders of the reproductive organs.

## Supplementary material

The supplementary material for this article can be found at http://doi.org/10.1017/S0033291725102602.
