# Supplementary material for: The overlooked link between reproductive system disorders and depression: a cohort study in 2 million women
Source: Psychol Med. 2025 Nov 21;55:e354. doi: 10.1017/S0033291725102602 (PMC13058623; doi:10.1017/S0033291725102602)
Supplement: Bliddal et al. supplementary material [file S0033291725102602sup001.docx]

Supplementary material to

The Overlooked Link Between Reproductive System Disorders and Depression: A Cohort Study in 2 Million Women

Authors: Bliddal M, Wesselhoeft R, Rasmussen L, Janecka M, Zaks N, Petersen LK, Egsgaard S, Jensen PB, Munk-Olsen T.

Table of Content

Supplementary Figure 1 2

Supplementary Figure 2 3

Supplementary Figure 3 5

Supplementary Figure 4 7

Supplementary Figure 5 9

Supplementary Figure 6 11

Supplementary Figure 7 13

Supplementary Table 1 15

Supplementary Table 2 19

**Supplementary Figure 1. Design diagram**

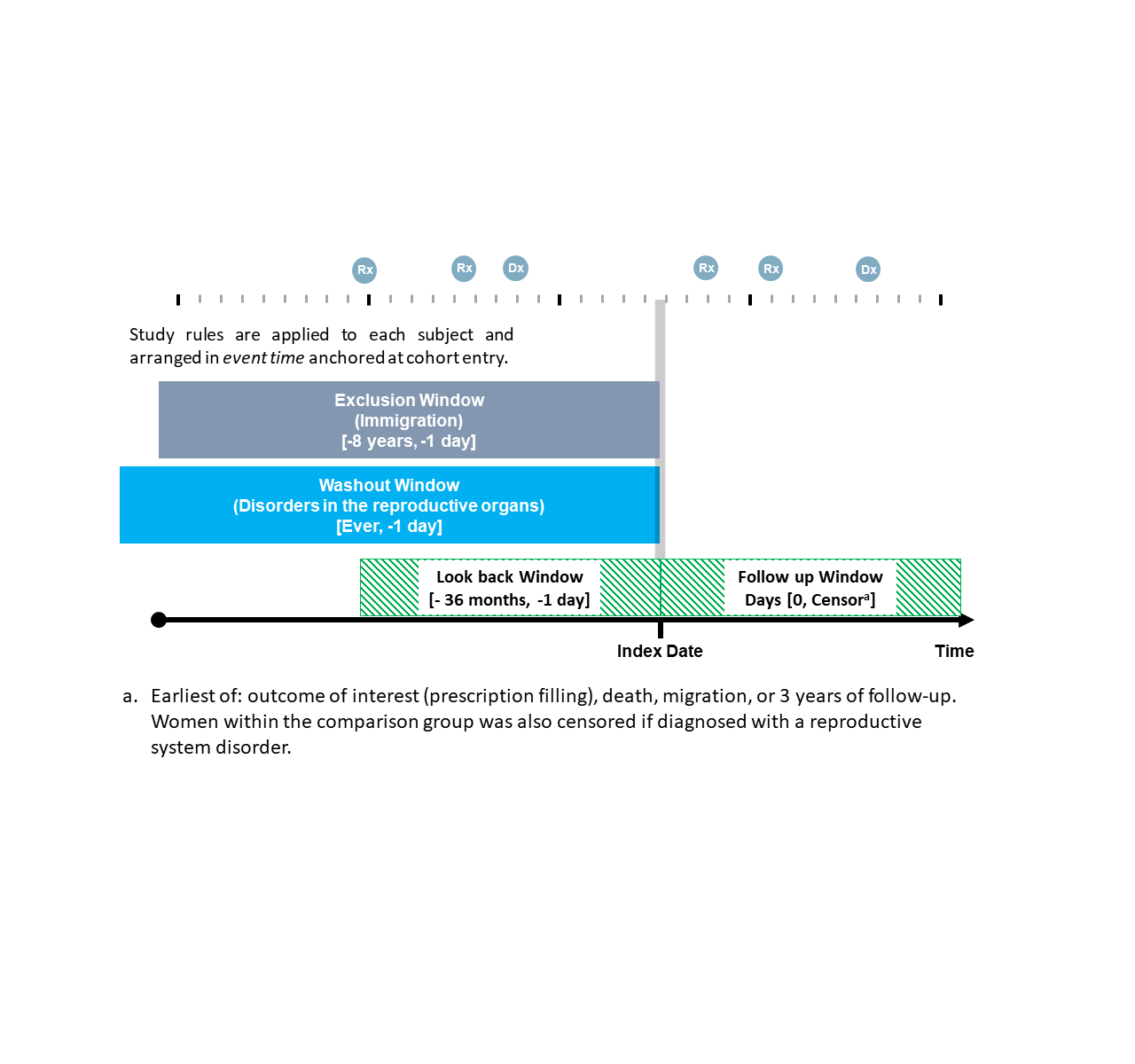


Source: Adapted from www.repeatinitiative.org/projects.html^20^

Design diagram on use of antidepressants in women aged 15-49 years with an incident diagnosis of a reproductive system disorder.

**Supplementary Figure 2.** **Incidence rate of depression (defined by ICD-10 codes F32-39 or ATC codes N06A) in quarterly intervals, 3 years before and after diagnosis of 15 reproductive system disorders (RSDs) (red), compared to age- and calendar matched comparators (blue).**

Incidence rate of depression (defined by ICD-10 codes F32-39 or ATC codes N06A) in quarterly intervals, 3 years before and after diagnosis of 15 reproductive system disorders (RSDs) (red), compared to age- and calendar matched comparators (blue). One panel is shown for composite reproductive disorders and for each RSD, presented in order of prevalence (the nine most common are reported in the main paper). Starting from upper right: Any RSD: ICD-10 E28, E70-E77, N80-N94; Other inflammatory (vagina/vulva): ICD-10 N76; Inflammatory uterine disorder: ICD-10 N71; Salpingitis and oophoritis: ICD-10 N70; Diseases of Bartholin’s gland: ICD-10 N75; Non-inflammatory vulva/perineum: ICD-10 N90; Scanty and rare menstruation: ICD-10 N91; Other non-inflammatory disorder vagina: ICD-10 N89; Other female pelvic inflammation: ICD-10 N73; Non-inflammatory disorder uterus, except cervix: ICD-10 N85; Inflammatory disorder of cervix uteri: ICD-10 N72; Non-inflammatory disorder of cervix: ICD-10 N88; Erosion/ectopia of cervix: ICD-10 N86; Fistula involving genital tract: ICD-10 N82; Vulvovaginal ulceration/inflammation: ICD-10 N77; Female pelvic inflammatory disorder: ICD-10 N74. The dotted lines indicate index date (day of incident RSD diagnosis).

**Supplementary Figure 3.** **Prevalence proportion of depression (defined by ICD-10 codes F32-39 or ATC codes N06A) in quarterly intervals, 3 years before and after diagnosis of a reproductive system disorder (RSD) (red), compared to age- and calendar matched comparators (blue).**

Prevalence proportion of depression (defined by ICD-10 codes F32-39 or ATC codes N06A) in quarterly intervals, 3 years before and after diagnosis of a reproductive system disorder (RSD) (red), compared to age- and calendar matched comparators (blue). One panel is shown for composite reproductive disorders and for each RSD presented in order of prevalence (the nine most common are reported in the main paper). Starting upper right corner: Any RSD: ICD-10 E28, E70-E77, N80-N94; Other inflammatory (vagina/vulva): ICD-10 N76; Inflammatory uterine disorder: ICD-10 N71; Salpingitis and oophoritis: ICD-10 N70; Diseases of Bartholin’s gland: ICD-10 N75; Non-inflammatory vulva/perineum: ICD-10 N90; Scanty and rare menstruation: ICD-10 N91; Other noninflmatory disorder vagina: ICD-10 N89; Other female pelvic inflammation: ICD-10 N73; Non-inflammatory disorder uterus, except cervix: ICD-10 N85; Inflammatory disorder of cervix uteri: ICD-10 N72; Non-inflammatory disorder of cervix: ICD-10 N88; Erosion/ectopia of cervix: ICD-10 N86; Fistula involving genitial tract: ICD-10 N82; Vulvovaginal ulceration/inflammation: ICD-10 N77; Female pelvic inflammation. Dis: ICD-10 N74. The dotted lines indicate index date (day of incident RSD diagnosis).

**Supplementary Figure 4.** **Incidence rate of depression (*defined by hospital diagnosis of depression (ICD-10 codes F32-39 or ATC codes N06A with a depression indication*) in quarterly intervals within 3 years before and after diagnosis of a reproductive system disorder (RSD) (red) and among age- and calendar matched comparators (blue).**

Incidence rate of depression (*defined by hospital diagnosis of depression (ICD-10 codes F32-39 or ATC codes N06A with a depression indication*) in quarterly intervals, 3 years before and after diagnosis of a reproductive system disorders (RSDs) (red), compared to age- and calendar matched comparators (blue). One panel for composite RSDs and for each RSD, presented in order of prevalence. Starting from upper right: Any RSD: ICD-10 E28, E70-E77, N80-N94; Abnormal menstruation: ICD-10 N92; Non-inflammatory diseases: ICD-10 N83; Dysplasia of cervix uteri: ICD-10 N87; Pain, reproductive system: ICD-10 N94; Poly of genital tract: ICD-10 N84; Other abnormal bleeding: ICD-10 N93; Endometriosis: ICD-10 N80; Genital prolapse: ICD-10 N81; Ovarian dysfunction including polycystic ovary syndrome: ICD-10 E28; Other inflammatory disorders (vagina/vulva): ICD-10 N76; Inflammatory uterine diseases: ICD-10 N71; Salpingitis and oophoritis: ICD-10 N70; Diseases of Bartholin’s gland: ICD-10 N75; Non-inflammatory diseases in vulva/perineum: ICD-10 N90; Scanty and rare menstruation: ICD-10 N91; Other non-inflammatory diseases of vagina: ICD-10 N89; Other female pelvic inflammatory diseases: ICD-10 N73; Non-inflammatory diseases of uterus or cervix: ICD-10 N85; Inflammatory diseases of cervix uteri: ICD-10 N72; Non-inflammatory diseases of cervix: ICD-10 N88; Erosion/ectopia of cervix: ICD-10 N86; Fistula involving genital tract: ICD-10 N82; Vulvovaginal ulceration/inflammation: ICD-10 N77; Female pelvic inflammatory disorders: ICD-10 N74. The dotted line indicate index date (day of incident RSD diagnosis).

**Supplementary Figure 5. Prevalence proportion of depression (*defined by ICD-10 codes F32-39 or ATC codes N06A with a depression indication*) in quarterly intervals, 3 years before and after diagnosis of a reproductive system disorder (RSD) (red), compared to age- and calendar matched comparators (blue).**

The prevalence proportion of depression (*defined by ICD-10 codes F32-39 or ATC codes N06A with a depression indication*) in quarterly intervals, 3 years before and after diagnosis of a reproductive system disorders (RSDs) (red), compared to age- and calendar matched comparators (blue). One panel for composite RSDs and for each RSD, presented in order of prevalence. Starting from upper right: Any RSD: ICD-10 E28, E70-E77, N80-N94; Abnormal menstruation: ICD-10 N92; Non-inflammatory diseases: ICD-10 N83; Dysplasia of cervix uteri: ICD-10 N87; Pain, reproductive system: ICD-10 N94; Poly of genital tract: ICD-10 N84; Other abnormal bleeding: ICD-10 N93; Endometriosis: ICD-10 N80; Genital prolapse: ICD-10 N81; Ovarian dysfunction including polycystic ovary syndrome: ICD-10 E28; Other inflammatory disorders (vagina/vulva): ICD-10 N76; Inflammatory uterine diseases: ICD-10 N71; Salpingitis and oophoritis: ICD-10 N70; Diseases of Bartholin’s gland: ICD-10 N75; Non-inflammatory diseases in vulva/perineum: ICD-10 N90; Scanty and rare menstruation: ICD-10 N91; Other non-inflammatory diseases of vagina: ICD-10 N89; Other female pelvic inflammatory diseases: ICD-10 N73; Non-inflammatory diseases of uterus or cervix: ICD-10 N85; Inflammatory diseases of cervix uteri: ICD-10 N72; Non-inflammatory diseases of cervix: ICD-10 N88; Erosion/ectopia of cervix: ICD-10 N86; Fistula involving genital tract: ICD-10 N82; Vulvovaginal ulceration/inflammation: ICD-10 N77; Female pelvic inflammatory disorders: ICD-10 N74. The dotted line indicate index date (day of incident RSD diagnosis).

**Supplementary Figure 6.** **Incidence rates of depression (*defined by hospital diagnosis of depression (ICD-10 codes F32-39*) in quarterly intervals, 3 years before and after diagnosis of a reproductive system disorder (RSD) (red), compared to age- and calendar matched comparators (blue).**

Incidence rates of depression (*defined by hospital diagnosis of depression (ICD-10 codes F32-39*) in quarterly intervals, 3 years before and after diagnosis of a reproductive system disorders (RSDs) (red), compared to age- and calendar matched comparators (blue). One panel for composite RSDs and for each RSD, presented in order of prevalence. Starting from upper right: Any RSD: ICD-10 E28, E70-E77, N80-N94; Abnormal menstruation: ICD-10 N92; Non-inflammatory diseases: ICD-10 N83; Dysplasia of cervix uteri: ICD-10 N87; Pain, reproductive system: ICD-10 N94; Poly of genital tract: ICD-10 N84; Other abnormal bleeding: ICD-10 N93; Endometriosis: ICD-10 N80; Genital prolapse: ICD-10 N81; Ovarian dysfunction including polycystic ovary syndrome: ICD-10 E28; Other inflammatory disorders (vagina/vulva): ICD-10 N76; Inflammatory uterine diseases: ICD-10 N71; Salpingitis and oophoritis: ICD-10 N70; Diseases of Bartholin’s gland: ICD-10 N75; Non-inflammatory diseases in vulva/perineum: ICD-10 N90; Scanty and rare menstruation: ICD-10 N91; Other non-inflammatory diseases of vagina: ICD-10 N89; Other female pelvic inflammatory diseases: ICD-10 N73; Non-inflammatory diseases of uterus or cervix: ICD-10 N85; Inflammatory diseases of cervix uteri: ICD-10 N72; Non-inflammatory diseases of cervix: ICD-10 N88; Erosion/ectopia of cervix: ICD-10 N86; Fistula involving genital tract: ICD-10 N82; Vulvovaginal ulceration/inflammation: ICD-10 N77; Female pelvic inflammatory disorders: ICD-10 N74. The dotted line indicate index date (day of incident RSD diagnosis).

**Supplementary Figure 7**. **Prevalence proportion of depression (*defined by a hospital diagnosis of an ICD-10 code F32-39*) in quarterly intervals, 3 years before and after diagnosis of a reproductive system disorder (RSD) (red), compared to age- and calendar matched comparators (blue).**

The prevalence proportion of depression (*defined by a hospital diagnosis of an ICD-10 code F32-39*) in quarterly intervals, 3 years before and after diagnosis of a reproductive system disorders (RSDs) (red), compared to age- and calendar matched comparators (blue). One panel for composite RSDs and for each RSD, presented in order of prevalence. Starting from upper right: Any RSD: ICD-10 E28, E70-E77, N80-N94; Abnormal menstruation: ICD-10 N92; Non-inflammatory diseases: ICD-10 N83; Dysplasia of cervix uteri: ICD-10 N87; Pain, reproductive system: ICD-10 N94; Poly of genital tract: ICD-10 N84; Other abnormal bleeding: ICD-10 N93; Endometriosis: ICD-10 N80; Genital prolapse: ICD-10 N81; Ovarian dysfunction including polycystic ovary syndrome: ICD-10 E28; Other inflammatory disorders (vagina/vulva): ICD-10 N76; Inflammatory uterine diseases: ICD-10 N71; Salpingitis and oophoritis: ICD-10 N70; Diseases of Bartholin’s gland: ICD-10 N75; Non-inflammatory diseases in vulva/perineum: ICD-10 N90; Scanty and rare menstruation: ICD-10 N91; Other non-inflammatory diseases of vagina: ICD-10 N89; Other female pelvic inflammatory diseases: ICD-10 N73; Non-inflammatory diseases of uterus or cervix: ICD-10 N85; Inflammatory diseases of cervix uteri: ICD-10 N72; Non-inflammatory diseases of cervix: ICD-10 N88; Erosion/ectopia of cervix: ICD-10 N86; Fistula involving genital tract: ICD-10 N82; Vulvovaginal ulceration/inflammation: ICD-10 N77; Female pelvic inflammatory disorders: ICD-10 N74. The dotted line indicate index date (day of incident RSD diagnosis).

**Supplementary Table 1. List of potential relevant ICD10 diagnoses for disorders related to female reproductive organs**

| **Disorder** |  | **Diagnosis** |
| --- | --- | --- |
| **Endocrine, nutritional and metabolic diseases** | |  |
| Ovarian dysfunction | | E28 |
|  | Estrogen excess | E28.0 |
|  | Androgen excess | E28.1 |
|  | Polycystic ovarian syndrome | E28.2 |
|  | Primary ovarian failure | E28.3 |
|  | Other ovarian dysfunction | E28.8 |
|  | Ovarian dysfunction, unspecified | E28.9 |
| **Diseases of the genitourinary system** | |  |
| Salpingitis and oophoritis | | N70 |
|  | Acute salpingitis and oophoritis | N70.0 |
|  | Chronic salpingitis and oophoritis | N70.1 |
|  | Salpingitis and oophoritis, unspecified | N70.9 |
| Inflammatory disease of uterus, except cervix | | N71 |
|  | Acute inflammatory disease of uterus | N71.0 |
|  | Chronic inflammatory disease of uterus | N71.1 |
|  | Inflammatory disease of uterus, unspecified | N71.9 |
| Inflammatory disease of cervix uteri | | N72 |
| Other female pelvic inflammatory diseases | | N73 |
|  | Acute parametritis and pelvic cellulitis | N73.0 |
|  | Chronic parametritis and pelvic cellulitis | N73.1 |
|  | Unspecified parametritis and pelvic cellulitis | N73.2 |
|  | Female acute pelvic peritonitis | N73.3 |
|  | Female chronic pelvic peritonitis | N73.4 |
|  | Female pelvic peritonitis, unspecified | N73.5 |
|  | Female pelvic peritoneal adhesions | N73.6 |
|  | Other specified female pelvic inflammatory diseases | N73.8 |
|  | Female pelvic inflammatory disease, unspecified | N73.9 |
| Female pelvic inflammatory disorders in diseases classified elsewhere | | N74 |
|  | Tuberculous infection of cervix uteri | N74.0 |
|  | Female tuberculous pelvic inflammatory disease | N74.1 |
|  | Female syphilitic pelvic inflammatory disease | N74.2 |
|  | Female gonococcal pelvic inflammatory disease | N74.3 |
|  | Female chlamydial pelvic inflammatory disease | N74.4 |
|  | Female pelvic inflammatory disorders in other diseases classified elsewhere | N74.8 |
| Diseases of the Bartholin gland | | N75 |
|  | Cyst of Bartholin gland | N75.0 |
|  | Abscess of Bartholin gland | N75.1 |
|  | Other diseases of Bartholin gland | N75.8 |
|  | Disease of Bartholin gland, unspecified | N75.9 |
| Other inflammation of vagina and vulva | | N76 |
|  | Acute vaginitis | N76.0 |
|  | Subacute and chronic vaginitis | N76.1 |
|  | Acute vulvitis | N76.2 |
|  | Subacute and chronic vulvitis | N76.3 |
|  | Abscess of vulva | N76.4 |
|  | Ulceration of vagina | N76.5 |
|  | Ulceration of vulva | N76.6 |
|  | Other specified inflammation of vagina and vulva | N76.8 |
| Vulvovaginal ulceration and inflammation in diseases classified elsewhere | | N77 |
|  | Ulceration of vulva in infectious and parasitic diseases classified elsewhere | N77.0 |
|  | Vaginitis, vulvitis and vulvovaginitis in infectious and parasitic diseases classified elsewhere | N77.1 |
|  | Vulvovaginal ulceration and inflammation in other diseases classified elsewhere | N77.8 |
| **Non-inflammatory disorders of female genital tract** | |  |
| Endometriosis | | N80 |
|  | Endometriosis of uterus | N80.0 |
|  | Endometriosis of ovary | N80.1 |
|  | Endometriosis of fallopian tube | N80.2 |
|  | Endometriosis of pelvic peritoneum | N80.3 |
|  | Endometriosis of rectovaginal septum and vagina | N80.4 |
|  | Endometriosis of intestine | N80.5 |
|  | Endometriosis in cutaneous scar | N80.6 |
|  | Other endometriosis | N80.8 |
|  | Endometriosis, unspecified | N80.9 |
| Female genital prolapse | | N81 |
|  | Female urethrocele | N81.0 |
|  | Cystocele | N81.1 |
|  | Incomplete uterovaginal prolapse | N81.2 |
|  | Complete uterovaginal prolapse | N81.3 |
|  | Uterovaginal prolapse, unspecified | N81.4 |
|  | Vaginal enterocele | N81.5 |
|  | Rectocele | N81.6 |
|  | Other female genital prolapse | N81.8 |
|  | Female genital prolapse, unspecified | N81.9 |
| Fistulae involving female genital tract | | N82 |
|  | Vesicovaginal fistula | N82.0 |
|  | Other female urinary-genital tract fistulae | N82.1 |
|  | Fistula of vagina to small intestine | N82.2 |
|  | Fistula of vagina to large intestine | N82.3 |
|  | Other female intestinal-genital tract fistulae | N82.4 |
|  | Female genital tract-skin fistulae | N82.5 |
|  | Other female genital tract fistulae | N82.8 |
|  | Female genital tract fistula, unspecified | N82.9 |
| Non-inflammatory disorders of ovary, fallopian tube and broad ligament | | N83 |
|  | Follicular cyst of ovary | N83.0 |
|  | Corpus luteum cyst | N83.1 |
|  | Other and unspecified ovarian cysts | N83.2 |
|  | Acquired atrophy of ovary and fallopian tube | N83.3 |
|  | Prolapse and hernia of ovary and fallopian tube | N83.4 |
|  | Torsion of ovary, ovarian pedicle and fallopian tube | N83.5 |
|  | Hematosalpinx | N83.6 |
|  | Hematoma of broad ligament | N83.7 |
|  | Other non-inflammatory disorders of ovary, fallopian tube and broad ligament | N83.8 |
|  | Non-inflammatory disorder of ovary, fallopian tube and broad ligament, unspecified | N83.9 |
| Polyp of female genital tract | | N84 |
|  | Polyp of corpus uteri | N84.0 |
|  | Polyp of cervix uteri | N84.1 |
|  | Polyp of vagina | N84.2 |
|  | Polyp of vulva | N84.3 |
|  | Polyp of other parts of female genital tract | N84.8 |
|  | Polyp of female genital tract, unspecified | N84.9 |
| Other non-inflammatory disorders of uterus, except cervix | | N85 |
|  | Endometrial glandular hyperplasia | N85.0 |
|  | Endometrial adenomatous hyperplasia | N85.1 |
|  | Hypertrophy of uterus | N85.2 |
|  | Subinvolution of uterus | N85.3 |
|  | Malposition of uterus | N85.4 |
|  | Inversion of uterus | N85.5 |
|  | Intrauterine synechiae | N85.6 |
|  | Hematometra | N85.7 |
|  | Other specified non-inflammatory disorders of uterus | N85.8 |
|  | Non-inflammatory disorder of uterus, unspecified | N85.9 |
| Erosion and ectropion of cervix uteri | | N86 |
| Dysplasia of cervix uteri | | N87 |
|  | Mild cervical dysplasia | N87.0 |
|  | Moderate cervical dysplasia | N87.1 |
|  | Severe cervical dysplasia, not elsewhere classified | N87.2 |
|  | Dysplasia of cervix uteri, unspecified | N87.9 |
| Other non-inflammatory disorders of cervix uteri | | N88 |
|  | Leukoplakia of cervix uteri | N88.0 |
|  | Old laceration of cervix uteri | N88.1 |
|  | Stricture and stenosis of cervix uteri | N88.2 |
|  | Incompetence of cervix uteri | N88.3 |
|  | Hypertrophic elongation of cervix uteri | N88.4 |
|  | Other specified non-inflammatory disorders of cervix uteri | N88.8 |
|  | Non-inflammatory disorder of cervix uteri, unspecified | N88.9 |
| Other non-inflammatory disorders of vagina | | N89 |
|  | Mild vaginal dysplasia | N89.0 |
|  | Moderate vaginal dysplasia | N89.1 |
|  | Severe vaginal dysplasia, not elsewhere classified | N89.2 |
|  | Dysplasia of vagina, unspecified | N89.3 |
|  | Leukoplakia of vagina | N89.4 |
|  | Stricture and atresia of vagina | N89.5 |
|  | Tight hymenal ring | N89.6 |
|  | Hematocolpos | N89.7 |
|  | Other specified non-inflammatory disorders of vagina | N89.8 |
|  | Non-inflammatory disorder of vagina, unspecified | N89.9 |
| Other non-inflammatory disorders of vulva and perineum | | N90 |
|  | Mild vulvar dysplasia | N90.0 |
|  | Moderate vulvar dysplasia | N90.1 |
|  | Severe vulvar dysplasia, not elsewhere classified | N90.2 |
|  | Dysplasia of vulva, unspecified | N90.3 |
|  | Leukoplakia of vulva | N90.4 |
|  | Atrophy of vulva | N90.5 |
|  | Hypertrophy of vulva | N90.6 |
|  | Vulvar cyst | N90.7 |
|  | Other specified non-inflammatory disorders of vulva and perineum | N90.8 |
|  | Non-inflammatory disorder of vulva and perineum, unspecified | N90.9 |
| Absent, scanty and rare menstruation | | N91 |
|  | Primary amenorrhea | N91.0 |
|  | Secondary amenorrhea | N91.1 |
|  | Amenorrhea, unspecified | N91.2 |
|  | Primary oligomenorrhoea | N91.3 |
|  | Secondary oligomenorrhoea | N91.4 |
|  | Oligomenorrhoea, unspecified | N91.5 |
| Excessive, frequent and irregular menstruation | | N92 |
|  | Excessive and frequent menstruation with regular cycle | N92.0 |
|  | Excessive and frequent menstruation with irregular cycle | N92.1 |
|  | Excessive menstruation at puberty | N92.2 |
|  | Ovulation bleeding | N92.3 |
|  | Excessive bleeding in the premenopausal period | N92.4 |
|  | Other specified irregular menstruation | N92.5 |
|  | Irregular menstruation, unspecified | N92.6 |
| Other abnormal uterine and vaginal bleeding | | N93 |
|  | Postcoital and contact bleeding | N93.0 |
|  | Other specified abnormal uterine and vaginal bleeding | N93.8 |
|  | Abnormal uterine and vaginal bleeding, unspecified | N93.9 |
| Pain and other conditions associated with female genital organs and menstrual cycle | | N94 |
|  | Mittelschmerz | N94.0 |
|  | Dyspareunia | N94.1 |
|  | Vaginismus | N94.2 |
|  | Premenstrual tension syndrome | N94.3 |
|  | Primary dysmenorrhea | N94.4 |
|  | Secondary dysmenorrhea | N94.5 |
|  | Dysmenorrhea, unspecified | N94.6 |
|  | Other specified conditions associated with female genital organs and menstrual cycle | N94.8 |
|  | Unspecified condition associated with female genital organs and menstrual cycle | N94.9 |
|  |  |  |

**Supplementary Table 2. Incidence and incidence rate ratio of depression (defined by ICD-10 codes F32-39 or ATC codes N06A) during a 24-month window, including 12 months before and after the date of incident diagnosis of selected female reproductive system disorders, among women with any reproductive system disorder and 15 specific reproductive system disorders, compared to an age-matched comparator group. Disorder are ordered by frequency.**

| **Disorders, ICD-10** | **Depression (N)** | **Risk time** | **IR*** | **IRR*** |
| --- | --- | --- | --- | --- |
| **Any reproductive system disorders** | |  |  |  |
| Comparator | 37,330 | 91,488 | 0.41 | 1.00 |
| Case | 11,082 | 17,056 | 0.65 | 1.59 (1.56-1.63) |
| **Other inflammation (vagina/vulva), N76** | |  |  |  |
| Comparator | 2063 | 4067 | 0.51 | 1.00 |
| Case | 650 | 686 | 0.95 | 1.87 (1.71-2.04) |
| **Inflammatory uterine disorder, N71** | |  |  |  |
| Comparator | 1408 | 2825 | 0.50 | 1.00 |
| Case | 445 | 487 | 0.91 | 1.83 (1.65-2.04) |
| **Salpingitis & oophoritis, N70** | |  |  |  |
| Comparator | 1523 | 2821 | 0.54 | 1.00 |
| Case | 457 | 504 | 0.91 | 1.68 (1.51-1.86) |
| **Disease of Bartholin’s gland, N75** | |  |  |  |
| Comparator | 1120 | 2261 | 0.50 | 1.00 |
| Case | 293 | 413 | 0.71 | 1.43 (1.26-1.63) |
| **Non-inflammatory vulva/perineum, N90** | |  |  |  |
| Comparator | 1134 | 2271 | 0.50 | 1.00 |
| Case | 289 | 405 | 0.71 | 1.43 (1.26-1.63) |
| **Scanty and rare menstruation, N91** | |  |  |  |
| Comparator | 963 | 2024 | 0.48 | 1.00 |
| Case | 302 | 358 | 0.84 | 1.77 (1.56-2.02) |
| **Other non-inflammatory dis. vagina, N89** | |  |  |  |
| Comparator | 572 | 1216 | 0.47 | 1.00 |
| Case | 159 | 220 | 0.72 | 1.54 (1.29-1.83) |
| **Other female pelvic inflammatory N73** | |  |  |  |
| Comparator | 576 | 1142 | 0.50 | 1.00 |
| Case | 180 | 197 | 0.92 | 1.81 (1.53-2.14) |
| **Non-inflammatory disorder uterus, excluding cervix, N85** | |  |  |  |
| Comparator | 498 | 991 | 0.50 | 1.00 |
| Case | 129 | 178 | 0.72 | 1.44 (1.19-1.75) |
| **Inflammatory dis. of cervix uteri, N72** | |  |  |  |
| Comparator | 314 | 578 | 0.54 | 1.00 |
| Case | 82 | 100 | 0.82 | 1.52 (1.19-1.93) |
| **Non-inflammatory disorder of cervix, N88** | |  |  |  |
| Comparator | 266 | 528 | 0.50 | 1.00 |
| Case | 56 | 98 | 0.57 | 1.13 (0.85-1.50) |
| **Erosion/ectopia of cervix, N86** | |  |  |  |
| Comparator | 214 | 510 | 0.42 | 1.00 |
| Case | 58 | 99 | 0.59 | 1.40 (1.04-1.86) |
| **Fistulae involving genital tract, N82** | |  |  |  |
| Comparator | 137 | 271 | 0.51 | 1.00 |
| Case | 60 | 49 | 1.23 | 2.43 (1.79-3.28) |
| **Vulvovaginal ulceration/inflammation N77** | | |  |  |
| Comparator | 120 | 208 | 0.58 | 1.00 |
| Case | 39 | 36 | 1.10 | 1.90 (1.32-2.73) |
| **Female pelvic inflammatory disorder, N74** | |  |  |  |
| Comparator | 32 | 67 | 0.48 | 1.00 |
| Case | 12 | 11 | 1.12 | 2.35 (1.21-4.57) |
| * Incidence rate, ** Incidence rate ratio | |  |  |  |
